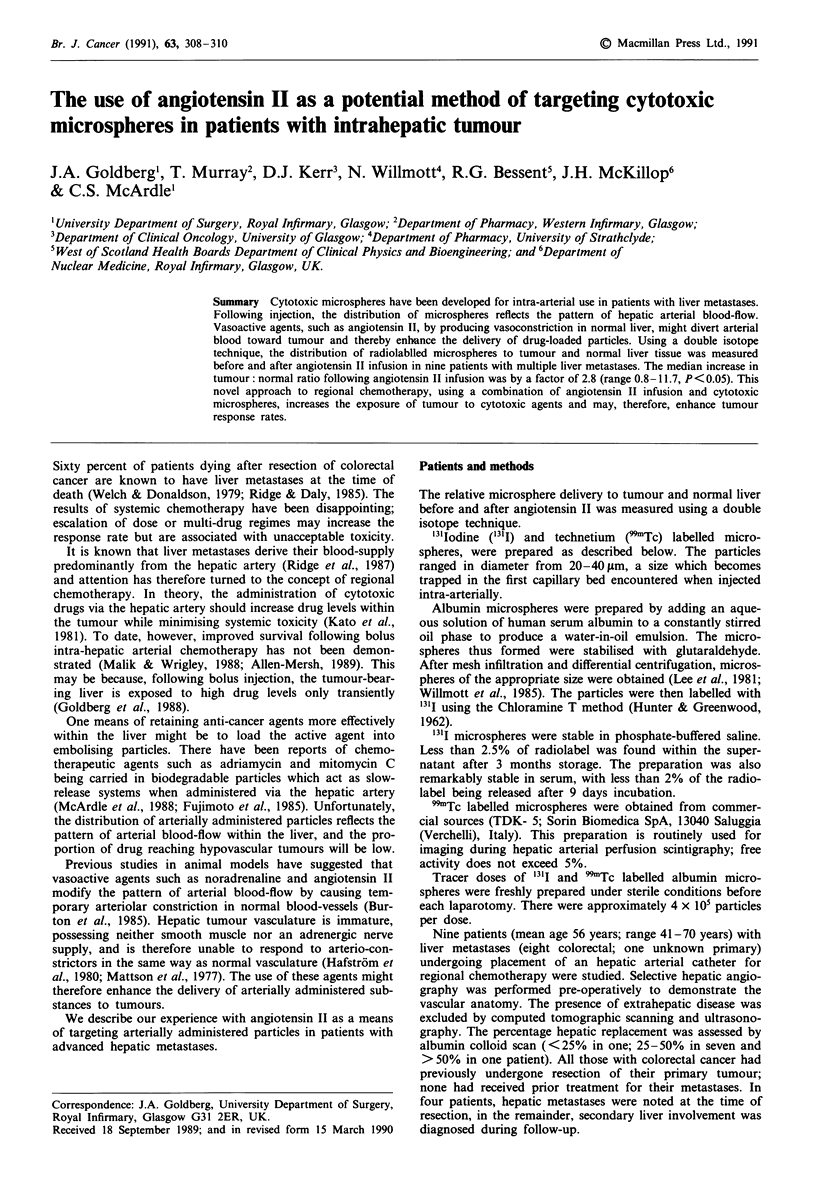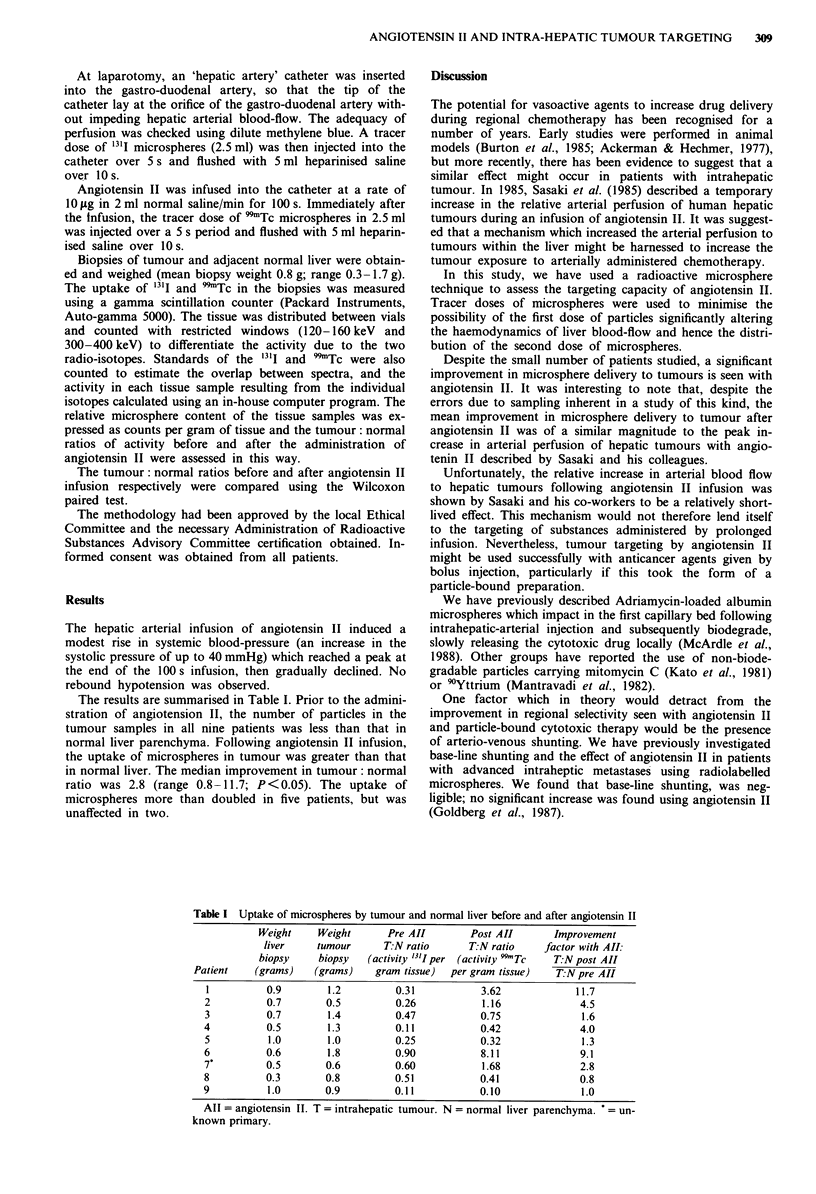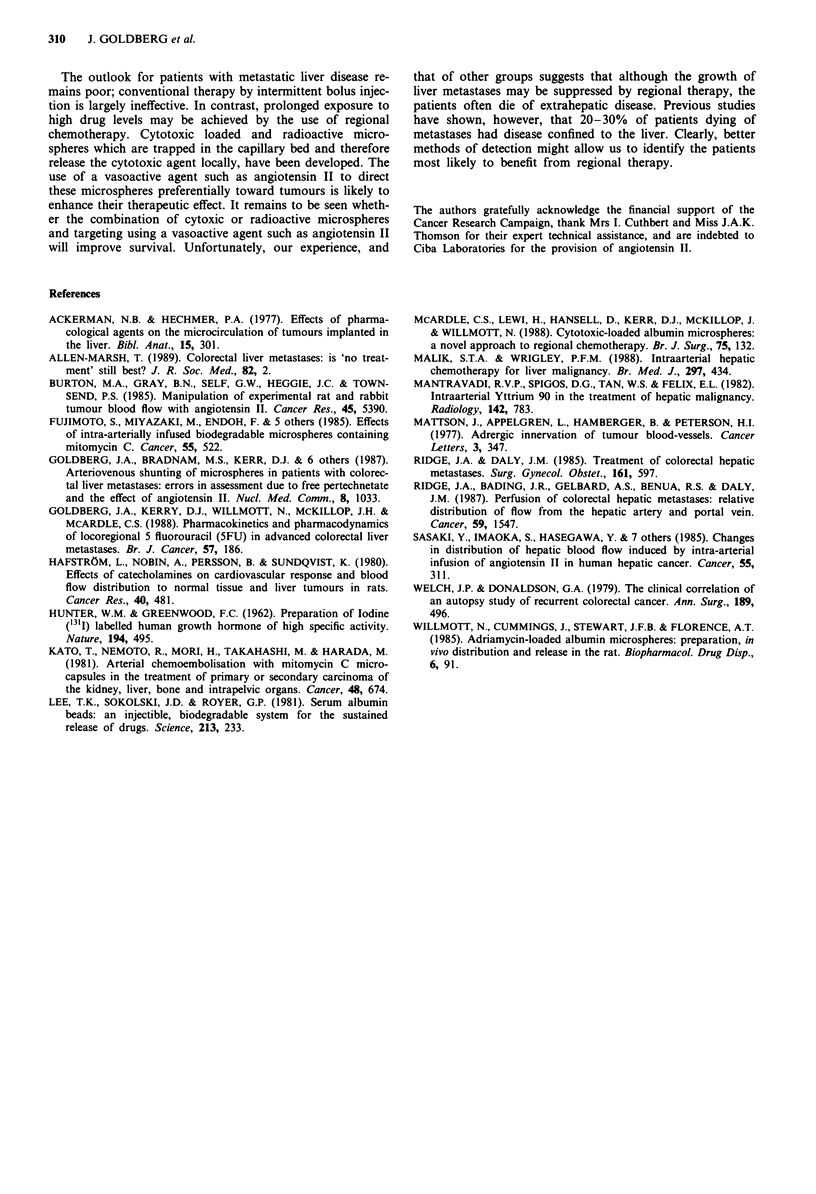# The use of angiotensin II as a potential method of targeting cytotoxic microspheres in patients with intrahepatic tumour.

**DOI:** 10.1038/bjc.1991.71

**Published:** 1991-02

**Authors:** J. A. Goldberg, T. Murray, D. J. Kerr, N. Willmott, R. G. Bessent, J. H. McKillop, C. S. McArdle

**Affiliations:** University Department of Surgery, Royal Infirmary, Glasgow, UK.

## Abstract

Cytotoxic microspheres have been developed for intra-arterial use in patients with liver metastases. Following injection, the distribution of microspheres reflects the pattern of hepatic arterial blood-flow. Vasoactive agents, such as angiotensin II, by producing vasoconstriction in normal liver, might divert arterial blood toward tumour and thereby enhance the delivery of drug-loaded particles. Using a double isotope technique, the distribution of radiolabelled microspheres to tumour and normal liver tissue was measured before and after angiotensin II infusion in nine patients with multiple liver metastases. The median increase in tumour: normal ratio following angiotensin II infusion was by a factor of 2.8 (range 0.8-11.7, P less than 0.05). This novel approach to regional chemotherapy, using a combination of angiotensin II infusion and cytotoxic microspheres, increases the exposure of tumour to cytotoxic agents and may, therefore, enhance tumour response rates.


Br. J. Cancer (1991), 63, 308-310 © Macmillan Press Ltd., 1991

### The use of angiotensin II as a potential method of targeting cytotoxic microspheres in patients with intrahepatic tumour

J.A. Goldberg[1], T. Murray[2], D.J. Kerr[3], N. Willmott[4], R.G. Bessent[5], J.H. McKillop[6] & C.S. McArdle[1]

[1]*University Department of Surgery, Royal Infirmary, Glasgow;* [2]*Department of Pharmacy, Western Infirmary, Glasgow;* [3]*Department of Clinical Oncology, University of Glasgow;* [4]*Department of Pharmacy, University of Strathclyde;* [5]*West of Scotland Health Boards Department of Clinical Physics and Bioengineering; and* [6]*Department of Nuclear Medicine, Royal Infirmary, Glasgow, UK.*

**Summary** Cytotoxic microspheres have been developed for intra-arterial use in patients with liver metastases. Following injection, the distribution of microspheres reflects the pattern of hepatic arterial blood-flow. Vasoactive agents, such as angiotensin II, by producing vasoconstriction in normal liver, might divert arterial blood toward tumour and thereby enhance the delivery of drug-loaded particles. Using a double isotope technique, the distribution of radiolablled microspheres to tumour and normal liver tissue was measured before and after angiotensin II infusion in nine patients with multiple liver metastases. The median increase in tumour : normal ratio following angiotensin II infusion was by a factor of 2.8 (range 0.8–11.7, $P < 0.05$). This novel approach to regional chemotherapy, using a combination of angiotensin II infusion and cytotoxic microspheres, increases the exposure of tumour to cytotoxic agents and may, therefore, enhance tumour response rates.

Sixty percent of patients dying after resection of colorectal cancer are known to have liver metastases at the time of death (Welch & Donaldson, 1979; Ridge & Daly, 1985). The results of systemic chemotherapy have been disappointing; escalation of dose or multi-drug regimes may increase the response rate but are associated with unacceptable toxicity.

It is known that liver metastases derive their blood-supply predominantly from the hepatic artery (Ridge *et al.*, 1987) and attention has therefore turned to the concept of regional chemotherapy. In theory, the administration of cytotoxic drugs via the hepatic artery should increase drug levels within the tumour while minimising systemic toxicity (Kato *et al.*, 1981). To date, however, improved survival following bolus intra-hepatic arterial chemotherapy has not been demonstrated (Malik & Wrigley, 1988; Allen-Mersh, 1989). This may be because, following bolus injection, the tumour-bearing liver is exposed to high drug levels only transiently (Goldberg *et al.*, 1988).

One means of retaining anti-cancer agents more effectively within the liver might be to load the active agent into embolising particles. There have been reports of chemotherapeutic agents such as adriamycin and mitomycin C being carried in biodegradable particles which act as slow-release systems when administered via the hepatic artery (McArdle *et al.*, 1988; Fujimoto *et al.*, 1985). Unfortunately, the distribution of arterially administered particles reflects the pattern of arterial blood-flow within the liver, and the proportion of drug reaching hypovascular tumours will be low.

Previous studies in animal models have suggested that vasoactive agents such as noradrenaline and angiotensin II modify the pattern of arterial blood-flow by causing temporary arteriolar constriction in normal blood-vessels (Burton *et al.*, 1985). Hepatic tumour vasculature is immature, possessing neither smooth muscle nor an adrenergic nerve supply, and is therefore unable to respond to arterio-constrictors in the same way as normal vasculature (Hafström *et al.*, 1980; Mattson *et al.*, 1977). The use of these agents might therefore enhance the delivery of arterially administered substances to tumours.

We describe our experience with angiotensin II as a means of targeting arterially administered particles in patients with advanced hepatic metastases.

Correspondence: J.A. Goldberg, University Department of Surgery, Royal Infirmary, Glasgow G31 2ER, UK.
Received 18 September 1989; and in revised form 15 March 1990

### Patients and methods

The relative microsphere delivery to tumour and normal liver before and after angiotensin II was measured using a double isotope technique.

$^{131}$Iodine ($^{131}$I) and technetium ($^{99m}$Tc) labelled microspheres, were prepared as described below. The particles ranged in diameter from 20–40 μm, a size which becomes trapped in the first capillary bed encountered when injected intra-arterially.

Albumin microspheres were prepared by adding an aqueous solution of human serum albumin to a constantly stirred oil phase to produce a water-in-oil emulsion. The microspheres thus formed were stabilised with glutaraldehyde. After mesh infiltration and differential centrifugation, microspheres of the appropriate size were obtained (Lee *et al.*, 1981; Willmott *et al.*, 1985). The particles were then labelled with $^{131}$I using the Chloramine T method (Hunter & Greenwood, 1962).

$^{131}$I microspheres were stable in phosphate-buffered saline. Less than 2.5% of radiolabel was found within the supernatant after 3 months storage. The preparation was also remarkably stable in serum, with less than 2% of the radiolabel being released after 9 days incubation.

$^{99m}$Tc labelled microspheres were obtained from commercial sources (TDK- 5; Sorin Biomedica SpA, 13040 Saluggia (Verchelli), Italy). This preparation is routinely used for imaging during hepatic arterial perfusion scintigraphy; free activity does not exceed 5%.

Tracer doses of $^{131}$I and $^{99m}$Tc labelled albumin microspheres were freshly prepared under sterile conditions before each laparotomy. There were approximately $4 \times 10^5$ particles per dose.

Nine patients (mean age 56 years; range 41–70 years) with liver metastases (eight colorectal; one unknown primary) undergoing placement of an hepatic arterial catheter for regional chemotherapy were studied. Selective hepatic angiography was performed pre-operatively to demonstrate the vascular anatomy. The presence of extrahepatic disease was excluded by computed tomographic scanning and ultrasonography. The percentage hepatic replacement was assessed by albumin colloid scan (<25% in one; 25–50% in seven and >50% in one patient). All those with colorectal cancer had previously undergone resection of their primary tumour; none had received prior treatment for their metastases. In four patients, hepatic metastases were noted at the time of resection, in the remainder, secondary liver involvement was diagnosed during follow-up.

At laparotomy, an 'hepatic artery' catheter was inserted into the gastro-duodenal artery, so that the tip of the catheter lay at the orifice of the gastro-duodenal artery without impeding hepatic arterial blood-flow. The adequacy of perfusion was checked using dilute methylene blue. A tracer dose of $^{131}$I microspheres (2.5 ml) was then injected into the catheter over 5 s and flushed with 5 ml heparinised saline over 10 s.

Angiotensin II was infused into the catheter at a rate of 10 µg in 2 ml normal saline/min for 100 s. Immediately after the infusion, the tracer dose of $^{99m}$Tc microspheres in 2.5 ml was injected over a 5 s period and flushed with 5 ml heparinised saline over 10 s.

Biopsies of tumour and adjacent normal liver were obtained and weighed (mean biopsy weight 0.8 g; range 0.3–1.7 g). The uptake of $^{131}$I and $^{99m}$Tc in the biopsies was measured using a gamma scintillation counter (Packard Instruments, Auto-gamma 5000). The tissue was distributed between vials and counted with restricted windows (120–160 keV and 300–400 keV) to differentiate the activity due to the two radio-isotopes. Standards of the $^{131}$I and $^{99m}$Tc were also counted to estimate the overlap between spectra, and the activity in each tissue sample resulting from the individual isotopes calculated using an in-house computer program. The relative microsphere content of the tissue samples was expressed as counts per gram of tissue and the tumour : normal ratios of activity before and after the administration of angiotensin II were assessed in this way.

The tumour : normal ratios before and after angiotensin II infusion respectively were compared using the Wilcoxon paired test.

The methodology had been approved by the local Ethical Committee and the necessary Administration of Radioactive Substances Advisory Committee certification obtained. Informed consent was obtained from all patients.

### Results

The hepatic arterial infusion of angiotensin II induced a modest rise in systemic blood-pressure (an increase in the systolic pressure of up to 40 mmHg) which reached a peak at the end of the 100 s infusion, then gradually declined. No rebound hypotension was observed.

The results are summarised in Table I. Prior to the administration of angiotension II, the number of particles in the tumour samples in all nine patients was less than that in normal liver parenchyma. Following angiotensin II infusion, the uptake of microspheres in tumour was greater than that in normal liver. The median improvement in tumour : normal ratio was 2.8 (range 0.8–11.7; $P < 0.05$). The uptake of microspheres more than doubled in five patients, but was unaffected in two.

### Discussion

The potential for vasoactive agents to increase drug delivery during regional chemotherapy has been recognised for a number of years. Early studies were performed in animal models (Burton *et al.*, 1985; Ackerman & Hechmer, 1977), but more recently, there has been evidence to suggest that a similar effect might occur in patients with intrahepatic tumour. In 1985, Sasaki *et al.* (1985) described a temporary increase in the relative arterial perfusion of human hepatic tumours during an infusion of angiotensin II. It was suggested that a mechanism which increased the arterial perfusion to tumours within the liver might be harnessed to increase the tumour exposure to arterially administered chemotherapy.

In this study, we have used a radioactive microsphere technique to assess the targeting capacity of angiotensin II. Tracer doses of microspheres were used to minimise the possibility of the first dose of particles significantly altering the haemodynamics of liver blood-flow and hence the distribution of the second dose of microspheres.

Despite the small number of patients studied, a significant improvement in microsphere delivery to tumours is seen with angiotensin II. It was interesting to note that, despite the errors due to sampling inherent in a study of this kind, the mean improvement in microsphere delivery to tumour after angiotensin II was of a similar magnitude to the peak increase in arterial perfusion of hepatic tumours with angiotenin II described by Sasaki and his colleagues.

Unfortunately, the relative increase in arterial blood flow to hepatic tumours following angiotensin II infusion was shown by Sasaki and his co-workers to be a relatively short-lived effect. This mechanism would not therefore lend itself to the targeting of substances administered by prolonged infusion. Nevertheless, tumour targeting by angiotensin II might be used successfully with anticancer agents given by bolus injection, particularly if this took the form of a particle-bound preparation.

We have previously described Adriamycin-loaded albumin microspheres which impact in the first capillary bed following intrahepatic-arterial injection and subsequently biodegrade, slowly releasing the cytotoxic drug locally (McArdle *et al.*, 1988). Other groups have reported the use of non-biodegradable particles carrying mitomycin C (Kato *et al.*, 1981) or $^{90}$Yttrium (Mantravadi *et al.*, 1982).

One factor which in theory would detract from the improvement in regional selectivity seen with angiotensin II and particle-bound cytotoxic therapy would be the presence of arterio-venous shunting. We have previously investigated base-line shunting and the effect of angiotensin II in patients with advanced intraheptic metastases using radiolabelled microspheres. We found that base-line shunting, was negligible; no significant increase was found using angiotensin II (Goldberg *et al.*, 1987).

**Table I** Uptake of microspheres by tumour and normal liver before and after angiotensin II

| *Patient* | *Weight liver biopsy (grams)* | *Weight tumour biopsy (grams)* | *Pre AII T:N ratio (activity $^{131}$I per gram tissue)* | *Post AII T:N ratio (activity $^{99m}$Tc per gram tissue)* | *Improvement factor with AII: T:N post AII / T:N pre AII* |
|---|---|---|---|---|---|
| 1 | 0.9 | 1.2 | 0.31 | 3.62 | 11.7 |
| 2 | 0.7 | 0.5 | 0.26 | 1.16 | 4.5 |
| 3 | 0.7 | 1.4 | 0.47 | 0.75 | 1.6 |
| 4 | 0.5 | 1.3 | 0.11 | 0.42 | 4.0 |
| 5 | 1.0 | 1.0 | 0.25 | 0.32 | 1.3 |
| 6 | 0.6 | 1.8 | 0.90 | 8.11 | 9.1 |
| 7* | 0.5 | 0.6 | 0.60 | 1.68 | 2.8 |
| 8 | 0.3 | 0.8 | 0.51 | 0.41 | 0.8 |
| 9 | 1.0 | 0.9 | 0.11 | 0.10 | 1.0 |

AII = angiotensin II. T = intrahepatic tumour. N = normal liver parenchyma. * = unknown primary.

The outlook for patients with metastatic liver disease remains poor; conventional therapy by intermittent bolus injection is largely ineffective. In contrast, prolonged exposure to high drug levels may be achieved by the use of regional chemotherapy. Cytotoxic loaded and radioactive microspheres which are trapped in the capillary bed and therefore release the cytotoxic agent locally, have been developed. The use of a vasoactive agent such as angiotensin II to direct these microspheres preferentially toward tumours is likely to enhance their therapeutic effect. It remains to be seen whether the combination of cytoxic or radioactive microspheres and targeting using a vasoactive agent such as angiotensin II will improve survival. Unfortunately, our experience, and that of other groups suggests that although the growth of liver metastases may be suppressed by regional therapy, the patients often die of extrahepatic disease. Previous studies have shown, however, that 20–30% of patients dying of metastases had disease confined to the liver. Clearly, better methods of detection might allow us to identify the patients most likely to benefit from regional therapy.

The authors gratefully acknowledge the financial support of the Cancer Research Campaign, thank Mrs I. Cuthbert and Miss J.A.K. Thomson for their expert technical assistance, and are indebted to Ciba Laboratories for the provision of angiotensin II.

### References

ACKERMAN, N.B. & HECHMER, P.A. (1977). Effects of pharmacological agents on the microcirculation of tumours implanted in the liver. *Bibl. Anat.*, **15**, 301.

ALLEN-MARSH, T. (1989). Colorectal liver metastases: is 'no treatment' still best? *J. R. Soc. Med.*, **82**, 2.

BURTON, M.A., GRAY, B.N., SELF, G.W., HEGGIE, J.C. & TOWNSEND, P.S. (1985). Manipulation of experimental rat and rabbit tumour blood flow with angiotensin II. *Cancer Res.*, **45**, 5390.

FUJIMOTO, S., MIYAZAKI, M., ENDOH, F. & 5 others (1985). Effects of intra-arterially infused biodegradable microspheres containing mitomycin C. *Cancer*, **55**, 522.

GOLDBERG, J.A., BRADNAM, M.S., KERR, D.J. & 6 others (1987). Arteriovenous shunting of microspheres in patients with colorectal liver metastases: errors in assessment due to free pertechnetate and the effect of angiotensin II. *Nucl. Med. Comm.*, **8**, 1033.

GOLDBERG, J.A., KERRY, D.J., WILLMOTT, N., MCKILLOP, J.H. & MCARDLE, C.S. (1988). Pharmacokinetics and pharmacodynamics of locoregional 5 fluorouracil (5FU) in advanced colorectal liver metastases. *Br. J. Cancer*, **57**, 186.

HAFSTRÖM, L., NOBIN, A., PERSSON, B. & SUNDQVIST, K. (1980). Effects of catecholamines on cardiovascular response and blood flow distribution to normal tissue and liver tumours in rats. *Cancer Res.*, **40**, 481.

HUNTER, W.M. & GREENWOOD, F.C. (1962). Preparation of Iodine ($^{131}$I) labelled human growth hormone of high specific activity. *Nature*, **194**, 495.

KATO, T., NEMOTO, R., MORI, H., TAKAHASHI, M. & HARADA, M. (1981). Arterial chemoembolisation with mitomycin C microcapsules in the treatment of primary or secondary carcinoma of the kidney, liver, bone and intrapelvic organs. *Cancer*, **48**, 674.

LEE, T.K., SOKOLSKI, J.D. & ROYER, G.P. (1981). Serum albumin beads: an injectible, biodegradable system for the sustained release of drugs. *Science*, **213**, 233.

MCARDLE, C.S., LEWI, H., HANSELL, D., KERR, D.J., MCKILLOP, J. & WILLMOTT, N. (1988). Cytotoxic-loaded albumin microspheres: a novel approach to regional chemotherapy. *Br. J. Surg.*, **75**, 132.

MALIK, S.T.A. & WRIGLEY, P.F.M. (1988). Intraarterial hepatic chemotherapy for liver malignancy. *Br. Med. J.*, **297**, 434.

MANTRAVADI, R.V.P., SPIGOS, D.G., TAN, W.S. & FELIX, E.L. (1982). Intraarterial Yttrium 90 in the treatment of hepatic malignancy. *Radiology*, **142**, 783.

MATTSON, J., APPELGREN, L., HAMBERGER, B. & PETERSON, H.I. (1977). Adrergic innervation of tumour blood-vessels. *Cancer Letters*, **3**, 347.

RIDGE, J.A. & DALY, J.M. (1985). Treatment of colorectal hepatic metastases. *Surg. Gynecol. Obstet.*, **161**, 597.

RIDGE, J.A., BADING, J.R., GELBARD, A.S., BENUA, R.S. & DALY, J.M. (1987). Perfusion of colorectal hepatic metastases: relative distribution of flow from the hepatic artery and portal vein. *Cancer*, **59**, 1547.

SASAKI, Y., IMAOKA, S., HASEGAWA, Y. & 7 others (1985). Changes in distribution of hepatic blood flow induced by intra-arterial infusion of angiotensin II in human hepatic cancer. *Cancer*, **55**, 311.

WELCH, J.P. & DONALDSON, G.A. (1979). The clinical correlation of an autopsy study of recurrent colorectal cancer. *Ann. Surg.*, **189**, 496.

WILLMOTT, N., CUMMINGS, J., STEWART, J.F.B. & FLORENCE, A.T. (1985). Adriamycin-loaded albumin microspheres: preparation, *in vivo* distribution and release in the rat. *Biopharmacol. Drug Disp.*, **6**, 91.